# Quadrigeminal arachnoid cyst with perinatal encephalocele

**DOI:** 10.1007/s00381-020-04626-2

**Published:** 2020-04-23

**Authors:** Kazuki Akutagawa, Goichiro Tamura, Takao Tsurubuchi, Eiichi Ishikawa, Akira Matsumura, Takayuki Inagaki

**Affiliations:** 1 Division of Pediatric Neurosurgery, Ibaraki Children’s Hospital, Mito, Ibaraki Japan; 2 Department of Neurosurgery, Faculty of Medicine, University of Tsukuba, 1-1-1 Tennodai, Tsukuba, Ibaraki Prefecture 305-8575 Japan; 3 Department of Neurosurgery, Faculty of Medicine, University of Tsukuba, Tsukuba, Ibaraki 305-8575 Japan

**Keywords:** Quadrigeminal arachnoid cyst, Endoscopic surgery, Cyst fenestration, Encephalocele

## Abstract

**Introduction:**

Quadrigeminal arachnoid cyst (QAC) associated with encephalocele is rare; and while some treatments have been developed in recent years, no definite therapeutic approach for QAC has been established. Endoscopic treatment for arachnoid cyst is gaining popularity because it is relatively less invasive to the normal brain tissues.

**Case presentation:**

The patient, a 4-year-old girl, presented with QAC associated with congenital occipital encephalocele. At the age of 1 month, repair of the perinatal encephalocele had been performed at another institute. An asymptomatic arachnoid cyst remained in the posterior fossa, which was closely monitored with follow up. At age 4 years, the patient started to complain of headache, which gradually increased in both strength and frequency. Magnetic resonance imaging (MRI) revealed cerebellar compression due to cyst enlargement. We performed neuroendoscopic cyst fenestration with an occipital bone approach. Post-operative MRI showed reduced size of the cyst, and the headache dramatically improved and resolved.

**Discussion:**

The standard treatment of QAC is still controversial; however, our successful use of endoscopic fenestration toward the third ventricle indicates its efficacy and safety. QACs have been classified into 3 types based on their expansion mechanisms; our case might suggest another possible mechanism of QAC development.

**Conclusion:**

In our case, endoscopic cyst fenestration was successful for QAC with perinatal encephalocele. However, long-term follow-up and analysis of similar cases are needed to determine its effectiveness.

## Introduction

Arachnoid cysts are pouch-like intra-arachnoid masses filled with cerebrospinal fluid (CSF). They account for 1% of all intracranial lesions and 2.6% of intracranial lesions among those aged below 18 years [1, 2]. Quadrigeminal cistern arachnoid cysts (QACs) are rare, accounting for 5–10% of all intracranial arachnoid cysts and tend to occur in young children [3–5]. QACs are often associated with congenital central nerve anomalies such as holoprosencephaly, Chiari malformation type II, and encephaloceles [6].

## Historical background

Several surgical procedures have been reported for QACs, including stereotactic aspiration, craniotomy and cyst excision or fenestration, ventriculoperitoneal or cystoperitoneal shunting, and combined procedures [7–10]. These procedures share a common goal, namely, to establish a discharge pathway for the CSF and change its dynamics. Recently, endoscopy has been considered as a better surgical option, because it enables the surgeon to fenestrate toward the conventional subarachnoid space while preserving normal arachnoid structure. This procedure is thought less invasive than open craniotomy and possibly avoids the complications caused by shunt treatment. Cyst-peritoneal shunt procedures carry some possibility of future shunt dependency and over drainage, but endoscopic surgery may avoid those complications. Many studies have discussed the role of endoscopic surgery in the treatment of intracranial arachnoid cysts, including some cases of QACs [6–8, 11–15].

Here, alongside our case report, we suggest possible mechanisms involved in the development of QACs and discuss the important role of neuroendoscopic surgery.

## Exemplary case description

### Clinical presentation

**Fig. 1:**
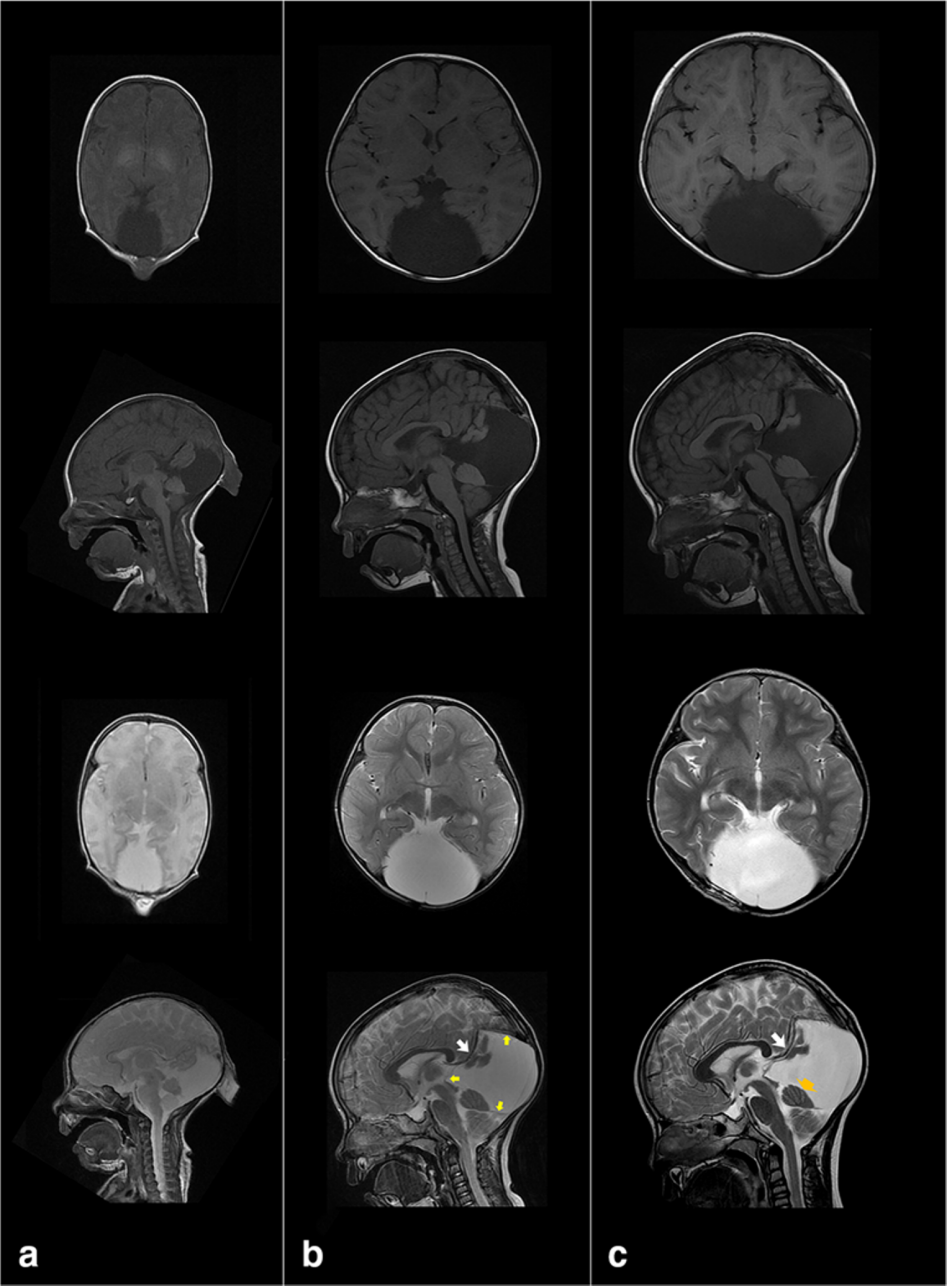
Preoperative MRI scan. **a** Axial and sagittal T1-weighted or T2-weighted image at birth showed the lack of cranium and encephalocele. Intracranial structures appear to protrude to outside of the cranium with the arachnoid membrane. **b** MRI at age 1 year and 10 months. Although subarachnoid cyst remained and gradually increased (small yellow arrow), there were no neurological symptoms. **c** MRI at age 4 years and 2 months. Axial and sagittal T1-weighted or T2-weighted image indicated enlargement of subarachnoid cyst not only horizontally but also vertically. The cyst compressed the bilateral cerebellar hemisphere from above (orange arrow). Sagittal T2-weighted image clearly shows an abnormal blood vessel continuous with the internal cerebral vein suggesting falcine sinus (white arrow, **b** and **c**)

The patient was a 4-year-old Asian girl, with a history of congenital encephalocele of the occipital region that was operated on at another hospital when she was 1 month old. The intraoperative findings after skin incision showed that there was a lack of galea on the defect of occipital bone that appeared as a small hole with a maximal diameter of 5 mm. The defect of the occipital bone was located near the midline occipital fissure. The intralumen of encephalocele sac contained encephalocele and stiff lipid-like tissues. The neck of the encephalocele sac seemed to continuously extend toward the subarachnoid space via the small defect of the galea and occipital bone. After ligation of the neck of the encephalocele, the ligated remnant tissue was automatically pulled down under the bone. Although the dura was not primarily sutured, the defect of the galea was sutured. An asymptomatic arachnoid cyst in the posterior fossa extending from quadrigeminal cistern to infratentorial supracerebellar space remained after the operation. During follow-up, imaging revealed which was closely monitored (Fig. 1 a and b). At 4 years old, gradually exacerbating headache and signs of delayed development appeared. Magnetic resonance imaging (MRI) showed cyst enlargement that was causing bilateral cerebellar hemisphere compression (Fig. 1c).

### Diagnosis

We made a diagnosis of enlarged QAC after the encephalocele removal.

### Management

**Fig. 2:**
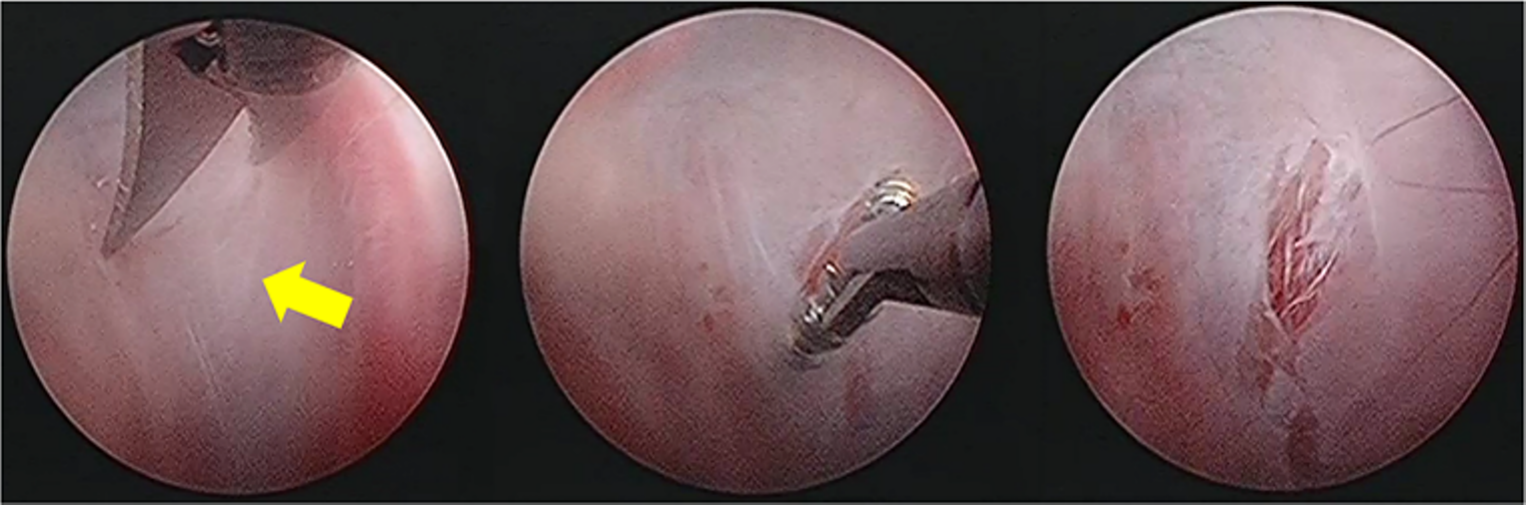
Intraoperative views. The rigid endoscope view showed the lumens of the cyst constituted multi-layer arachnoid membranes. We fenestrated them at 3 points toward the third ventricle (yellow arrow) and enlarge the hole of the arachnoid cyst and confirmed the pulsatile stream of CSF

We performed endoscopic cyst fenestration under general anesthesia. We made 1 burr hole on the left side of the posterior bone and used a rigid endoscope to carefully observe the intracystic lumen. The cyst membrane was visible just below the dura, and we were able to directly approach the normal subarachnoid space through the cyst lumen. There were multi-layers of arachnoid membranes without any sign of previous hemorrhage, which we fenestrated toward the conventional subarachnoid space, and we confirmed the pulsatile to-and-fro movement of the CSF (Fig. 2). The pathology report of the resected membrane showed normal arachnoid tissue.

### Prognosis and outcomes

**Fig. 3:**
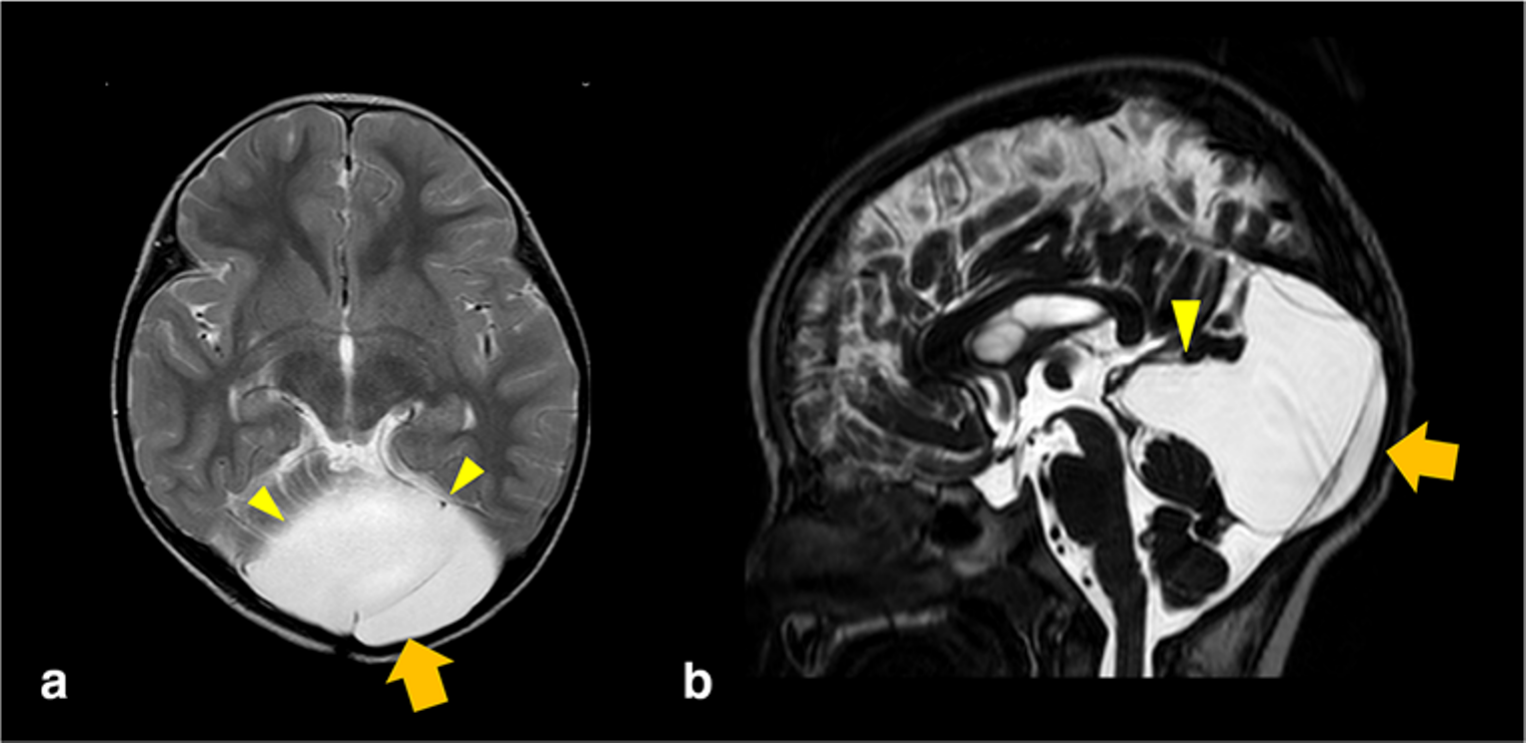
Postoperative MRI scan. **a** Axial T2-weighted image and **b** sagittal T2-weighted restored pulse image showing reduction of QAC (small yellow triangle). Rigid neuroendoscope inserted from left side of the posterior bone (large orange arrow)

After the operation, the headache subsided and eventually disappeared. One year after surgery, the headache did not recur, and MRI showed no further cyst enlargement (Fig. 3).

## Discussion

Arachnoid cyst is often associated with congenital abnormalities; a retrospective analysis showed that 25.41% of patients with arachnoid cyst had some anomaly when they were born [3]. Some patients also have other central nervous system lesions such as holoprosencephaly, porencephaly, Chiari malformation type II, and encephaloceles [6]. Arachnoid cysts most likely originate from a minor aberration in the development of the arachnoid layer that leads to splitting or duplication of the membrane [16].

There are four hypothesis regarding the mechanism of arachnoid cyst development, which are still being debated: (1) a ball-valve mechanism; (2) an osmotic gradient between the intra- and extra-cystic medium; (3) primary malformation of the arachnoid membrane or cerebral lobe agenesis; and (4) fluid hypersecretion by the lining cells of the cyst wall. Cine MRI or endoscopic procedure can be used to visualize unidirectional pulsation of the CSF flow [17, 18]. In our case, considering the pathology results, it seems likely that the arachnoid membrane thickens during recovery from surgical invasion and that hypersecretion and malabsorption of the CSF occurred due to changes in the arachnoid membrane brought on by the primary surgery.

QACs appear close to the dorsal midbrain and compress midbrain aqueduct at an early stage of development. As a result, symptoms such as hydrocephalus (head circumference expansion, headache, vomiting, lifelessness, and choked disc), paralysis of upward gaze, and visual deficit can occur. Posterior cranial fossa arachnoid cysts cause dizziness, nystagmus, nausea, headache, mental deficiency, and gait disturbance. In our case, the patient had escalatory headache and a history of encephalocele, which led to the final diagnosis.

**Fig. 4:**
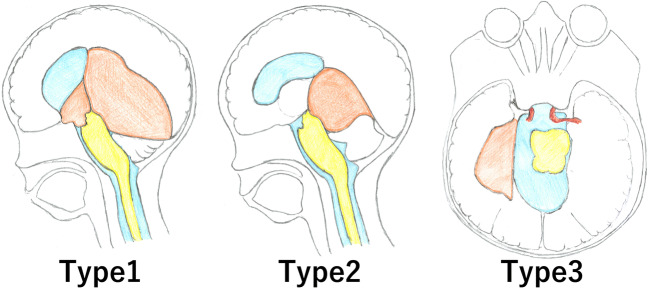
Classification the QACs expansion mechanism. Type 1: extension to both the supratentorial (at the level of the trigone) and the infratentorial regions (in the supracerebellar cistern). Type 2: only infratentorial extension in the supracerebellar or supraretrocerebellar regions. Type 3: lateral extension in the ambient cisterns toward the temporal lobe

A prospective study classified the QAC into three types based on their expansion mechanisms [17]:

Type 1: The most common type, with extension to both the supratentorial (at the level of the trigone) and infratentorial regions (in the supracerebellar cistern);
Type 2: A rarer type, with infratentorial extension to the supracerebellar or supraretrocerebellar regions; and
Type 3: Which has lateral extension to the ambient cisterns toward the temporal lobe (Fig. 4) [7].

In our case, the arachnoid cyst did not conform to any these three extension types; rather, it extended from dorsal cerebellar region for compressing cerebellar hemisphere. Compared with QAC type 2, which tend to extend toward anterior supracerebellar regions, the cyst in our case extended toward the posterior supracerebellar space. Generally, encephalocele forces the arachnoid membrane to extricate from the intracranial space and resulting in malformation. In our case, intraoperative findings showed that the outer arachnoid membrane was intact. We hypothesize that, in our case, an arachnoid cyst in posterior cranial space remained after the encephalocele operation, then, CSF malabsorption occurred in the arachnoid membrane resulting in the slow extension of the cyst. This mechanism is different from other congenital neurological abnormalities, such as holoprosencephaly and Chiari malformation type II, that do not involve extrication of the arachnoid membrane.

Multiple surgical strategies have been developed for the management of QACs. These include endoscopic resection of the cyst wall with establishment of continuous communication between the cyst lumen and the normal surrounding CSF pathways (ventricles or basal cisterns); craniotomy with microsurgical fenestration; and cyst-peritoneal shunting. Endoscopic treatment of QACs has been reported in retrospective studies, which have include isolated larger series’ and case reports, with encouraging results, especially when double fenestrations have been performed [7–10]. For treatment of the QAC in our case, we chose 3 spot fenestrations toward the conventional subarachnoid space with support from a rigid endoscope. We chose this approach for its relative simplicity and minimally invasive nature and its association with a lower morbidity and mortality than craniotomy. Endoscopic third ventriculostomy (ETV) is usually performed from burr hole made anterior to the coronal suture [4, 11]; however, Ruge et al. [14] reported a neuroendoscopic approach via a suboccipital supracerebellar approach, and Hopf and Perneczky et al. [19] reported endoscope-assisted microneurosurgery via a suboccipital craniotomy.

In our patient, we performed cyst fenestration toward the third ventricle via a suboccipital supracerebellar approach, because, prior to the procedure, we confirmed by MRI that there were no vital structures between dura and cyst membrane, such as brain tissue, arteries, or venous sinus. We chose a posterior approach to access the cyst lumen because it was easier and less invasive for the surrounding normal cerebral tissue than frontal ETV. The post-operative course has been good; however, we are not yet able to report long-term outcomes. Furthermore, it may be that the procedure used in our patient may be suitable only in particular cases; therefore, further studies are needed to verify the long-term prognosis and to analyze a larger number of similar cases.

## Conclusion

There are few reports of QAC associated with encephalocele. The uniqueness of the QAC in our case suggest there is a possibly another type of expanding of arachnoid cysts. We performed neuroendoscopic multiple fenestration via a suboccipital supracerebellar approach because this procedure is simple, minimally invasive, and associated with low morbidity and mortality. In our case, the prognosis is good, but long-term follow-up and the accumulation of similar cases are necessary to assess the applicability of this procedure.
